# Sustained activation of mTORC1 in macrophages increases AMPKα-dependent autophagy to maintain cellular homeostasis

**DOI:** 10.1186/s12858-016-0069-6

**Published:** 2016-07-07

**Authors:** Hongjie Pan, Xiao-ping Zhong, Sunhee Lee

**Affiliations:** Human Vaccine Institute and Department of Medicine, Duke University, Durham, NC 27710 USA; Department of Pediatrics-Allergy and Immunology, Duke University Medical Center, Durham, NC 27710 USA

**Keywords:** AMP-activated protein kinase (AMPK), Autophagy, Mechanistic target of rapamycin (mTOR), *Mycobacterium tuberculosis*, Macrophages, Tuberous sclerosis 1 (TSC1)

## Abstract

**Background:**

The mechanistic target of rapamycin complex 1 (mTORC1) is a well-conserved serine/threonine protein kinase that controls autophagy as well as many other processes such as protein synthesis, cell growth, and metabolism. The activity of mTORC1 is stringently and negatively controlled by the tuberous sclerosis proteins 1 and 2 complex (TSC1/2).

**Results:**

In contrast to the previous studies using *Tsc1* knockout mouse embryonic fibroblasts (MEF) cells, we demonstrated evidence that TSC1 deficient macrophages exhibited enhanced basal and mycobacterial infection-induced autophagy via AMPKα-dependent phosphorylation of ULK1 (Ser555). These effects were concomitant with constitutive activation of mTORC1 and can be reversed by addition of amino acids or rapamycin, and by the knockdown of the regulatory-associated protein of mTOR, Raptor. In addition, increased autophagy in TSC1 deficient macrophages resulted in suppression of inflammation during mycobacterial infection, which was reversed upon amino acid treatment of the TSC1 deficient macrophages. We further demonstrated that TSC1 conditional knockout mice infected with *Mycobacterium tuberculosis*, the causative agent of tuberculosis, resulted in less bacterial burden and a comparable level of inflammation when compared to wild type mice.

**Conclusions:**

Our data revealed that sustained activation of mTORC1 due to defects in TSC1 promotes AMPKα-dependent autophagic flux to maintain cellular homeostasis.

## Background

Autophagy is a fundamental and phylogenetically conserved self-degradation process that is characterized by the formation of double-layered vesicles (autophagosomes) around intracellular cargo for lysosomal delivery and proteolytic degradation [1, 2]. Autophagy is not only involved in cellular processes like nutrient regeneration and protein and organelle degradation, but also in clearance of intracellular pathogens, such as *M. tuberculosis* [3, 4]. Recent studies suggest that induction of autophagy in macrophages is an effective mechanism to enhance intracellular killing of *M. tuberculosis*, and that the ability of the pathogen to inhibit this process is of paramount importance for its survival [5, 6]. Autophagy is highly inducible via starvation, rapamycin, infection (virus, intracellular bacteria) and other cellular and environmental cues [2, 6]. The autophagosomal lipidated microtubule-associated protein 1 light chain 3 (LC3B-II) and the autophagy substrate sequestosome p62/SQSTM1 are often used to monitor autophagy [7, 8].

The best characterized regulator of autophagy is mTOR complex 1 (mTORC1). The rapamycin-sensitive mTORC1 kinase is composed of mTOR, regulatory-associated protein of mTOR (Raptor), mammalian lethal with SEC13 protein 8 (MLST8), the 40-kDa proline-rich Akt substrate (PRAS40), and DEP domain-containing mTOR-interacting protein (DEPTOR) [9]. Activation of mTORC1 depends on on the Ras-related GTPases (Rags) and Ras homolog enriched in brain (Rheb) GTPase and requires signals from amino acids, glucose, oxygen, energy (ATP), and growth factors (including cytokines and hormones such as insulin) [10].

mTORC1 positively regulates cell growth and proliferation by promoting many anabolic processes, including biosynthesis of proteins, lipids and organelles, and by limiting catabolic processes such as autophagy [11]. mTORC1 promotes protein synthesis by phosphorylating the eukaryotic initiation factor 4E (eIF4E)-binding protein 1 (4E-BP1) and the p70 ribosomal S6 kinase 1 (S6K1) [12, 13]. The phosphorylation of 4E-BP1 inhibits its binding to eIF4E, enabling eIF4E to promote translation [14]. The stimulation of S6K1 activity by mTORC1 leads to increases in mRNA biogenesis, translation and elongation, and the translation of ribosomal proteins through regulation of the activity of many proteins such as ribosomal protein S6 [15]. The mTORC1 kinase negatively regulates autophagy by inhibiting the activity of the UNC-51-like kinase 1 (ULK1, a yeast Atg1 homolog) through direct phosphorylation. mTORC1 activity depends on diverse positive signals such as high energy levels, normoxia, amino acids, and growth factors, all of which lead to the inhibition of autophagy [16]. Conversely, mTORC1 is inhibited when amino acids are scarce and growth factor signalling is reduced, and/or ATP concentrations fall, which results in activation of autophagy [17]. In addition, autophagy is regulated by AMP-activated protein kinase (AMPKα) that is widely recognized as a ubiquitous sensor of cellular energy status and regulates cellular metabolism to maintain energy homeostasis [18, 19]. Unlike the dependence of mTORC1 on various signals, AMPKα is activated only under low energy conditions. Under low nutrient conditions, AMPKα promotes autophagy by directly activating ULK1 through phosphorylation of Ser317, Ser555, and Ser777 [16, 20]. Under nutrient sufficiency, the active mTORC1 suppresses autophagy by phosphorylating ULK1 at Ser757 and affecting interaction between ULK1 and AMPKα [20, 21]. This coordinated phosphorylation is essential for ULK1 in autophagy induction.

One of the most important sensors involved in the regulation of mTORC1 activity is the tuberous sclerosis complex (TSC), which is a heterodimer comprised of TSC1 (also known as hamartin) and TSC2 (also known as tuberin) [11]. TSC1/2 works as a GTPase-activating protein (GAP) for Rheb. The active, GTP-bound form of Rheb directly interacts with mTORC1 to stimulate its activity [22, 23]. TSC1 is known to stabilize TSC2 by forming a complex with TSC2 [24, 25] and the genetic data strongly implicate the complex between TSC1 and TSC2 as the functional unit for these tumour suppressors [24].

Based on the current knowledge of autophagy regulation by mTORC1 [26], our initial hypothesis was that sustained activation of mTOR impairs autophagy in TSC1^fl/fl^ LysM-Cre^+^ mice and thus, TSC1^fl/fl^ LysM-Cre^+^ mice infected with an intracellular human pathogen, *Mycobacterium tuerculosis*, would have a significant bacterial burdens and would succumb to *M. tuberculosis* infection. To our surprise, the TSC1^fl/fl^ LysM-Cre^+^ mice infected with *M. tuberculosis* showed fewer bacteria in both spleens and lungs at the peak of infection. Further in vivo and in vitro analysis reveals that the unexpected autophagy dysfunction associated with loss of TSC1 in macrophages. Our results demonstrates that the complex interplay among mTORC1, AMPKα, and ULK1, and further highlights the importance of autophagy to support the cellular homeostasis during host-mycobacteria interactions.

## Results

### Sustained activation of mTORC1 via loss of TSC1 led to enhanced accumulation of autophagic markers

**Fig. 1:**
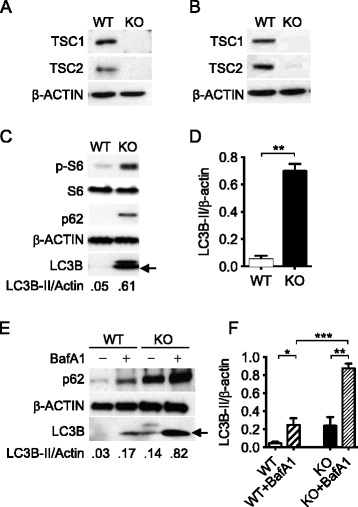
Loss of TSC1 led to sustained activation of the mTORC1 and to increased auto-phagosome formation. Representative Western blot of TSC1 and TSC2 in bone marrow macrophages from TSC1^f/f^-ERCre^+^ mice (**a**) and in the resident peritoneal macrophages of TSC1^fl/fl^ LysM-Cre^+^ mice (**b**) did not detect the TSC1 and TSC2 proteins. Isolated cells from mice were grown in 15 % L929 conditional medium for 4 days. 4-Hydroxytamoxifen at 2.5 mM was added into L929 conditioned culture medium for 2–3 days before use and the protein lysates were prepared. **c** Immunoblot showed significantly increased basal p-S6, LC3B-II (*arrow*), and p62 proteins in macrophages isolated from TSC1 KO mice. **d** Densitometric quantification of LC3-II (*n* = 3) band intensities quantified by ImageJ was normalized to total Actin. **e** TSC1 WT and KO BMMϕ were treated with 100 nM bafilomycin A1 for 3 h to measure autophagic flux. **f** Densitometric quantification of LC3-II (*n* = 3) was normalized to total Actin. The experiments were repeated three times. The arrow indicates LC3B-II. BafA1: bafilomycin A1. *, *p* <0.05, **, *p* < 0.01, ***, *p* < 0.001

Previous studies demonstrated that TSC1 deficient bone marrow derived macrophages (BMMϕ) constitutively activated mTORC1 [27]. To determine how TSC1 deficiency affects autophagy, TSC1^f/f^ LysM-Cre^+^ mice and TSC1^f/f^-ERCre^+^ mice were used in in vivo and in vitro studies respectively. Since LysMcre mice allow for both specific and highly efficient Cre–mediated deletion of loxP–flanked target genes in myeloid cells [28] and *M. tuberculosis* resides in *macrophages* during lung infection, we used TSC1^f/f^LysM-Cre^+^ mice for in vivo studies. For in vitro studies, we used tamoxifen-dependent Cre recombinases, so-called CreER recombinases, to generate time- and tissue-specific mouse mutants [29]. TSC1^f/f^-ERCre^+^ BMMϕ were in vitro differentiated for 3 days [27], at which time BMMϕ size was similar to controls. Here TSC1 was not detected in both tamoxifen-treated BMMϕ from TSC1^f/f^-ERCre^+^ mice (Fig. 1a) and peritoneal macrophages from TSC1^f/f^ LysM-Cre^+^ mice (Fig. 1b). TSC2 was also diminished in both macrophages, which indicates that TSC1 stabilizes TSC2 in macrophages and other cell types [30].

As compared with macrophages from wild type control mice, BMMϕ from TSC1 KO demonstrated increased mTORC1 activation as shown by elevated phosphorylation of the mTORC1 downstream target S6 protein (Fig. 1c). Surprisingly, higher levels of LC3B-II were observed in TSC1 KO macrophages compared to WT counterparts, which is in contrast to the current knowledge that mTORC1 activation by TSC1/2 loss inhibits autophagy [31, 32]. A reduction in LC3B-II was observed in TSC1 and TSC2 KO mouse embryonic fibroblasts (MEFs) [33–35]. However, similar to the MEFs, TSC1 KO macrophages had increased levels of the autophagy substrate p62 (Fig. 1c).

To evaluate autophagic flux in TSC1 KO macrophages, both TSC1 WT and KO BMMϕ cells were treated with bafilomycin A1, an inhibitor of autophagic degradation that prevents fusion between autophagosomes and lysosomes (Fig. 1e, f). Enchanced LC3B and p62 protein levels indicates presence of autophagic flux while no change indicates inhibition of autophagic degradation. As shown in Fig. 1e and f, both LC3B and p62 proteins substantially accumulated after bafilomycin A1 treatment, indicating that there is ongoing autophagic flux in the TSC1 KO macrophages. In addition, the ratio of LC3-II of bafilomycin treated and untreated macrophages (BafA1 treated/untreated) indicated that, TSC1 KO macrophages had a similar level of autophagic flux compared with wild type control (*p* = 0.7, data not shown). This result again confirms that mTORC1 activity did not affect the LC3-II transit through the autophagy pathway. Taken together, the the accumulation of autophagic markers and autophagic activity in the TSC1 KO macrophages suggest that the autophagy regulation in response to reduced TSC1 function significantly differs among various cell types.

### Suppression of mTORC1 activation in TSC1 KO macrophages resulted in reduced autophagic activity

**Fig. 2:**
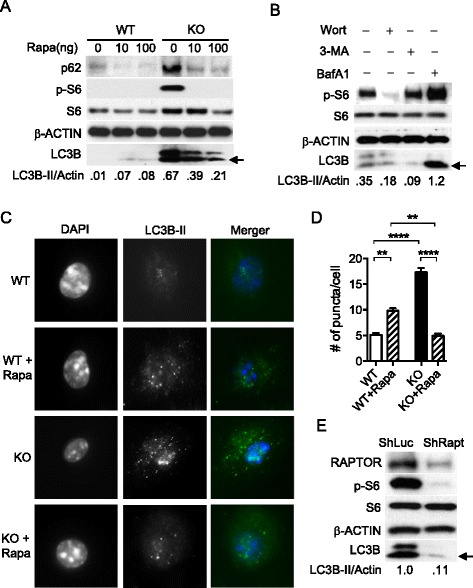
Autophagy was increased upon inhibition of the mTOR-dependent autphagic pathway in wild type macrophages as expected, but not in TSC1 KO macrophages. **a** BMMϕ were treated with the mTOR inhibitor, rapamycin at 0, 10, 100 ng/ml for 24 h, and then lysed for Western blot analysis. **b** TSC1 KO BMMϕ were treated with autophagy inhibitors, 5 mM of 3-methyladenine, 1 μM of wortmannin, and 100 μM of bafilomycin A1 for 2 h. **c** Autophagic puncta were visulized using immunofluorescent microscopy (1,000X magnification). TSC1 WT and KO BMMϕ were grown in chamber slides and treated with rapamycin at 20 ng/ml overnight. **d** The number of puncta was counted in at least 20 cells for each group under the fluorescent microscope and average number of puncta per cells was calculated. **e** TSC1 KO BMMϕ were transfected with shLUC (control) or shRAPTOR lentivirus and selected in the presence of puromycin at 2 μg/ml for 4 days and Western blot analysis was performed with indicated antibodies including p-AMPKα (T172). All the experiments were repeated at least three times. ImageJ was used to quantify band intensities and the ratio of LC3B-II/Actin (loading control) is shown. The arrow indicates LC3B-II. Rapa, rapamycin; Un, uninfected; WT, Wild type; KO, knockout; ShLUC, shRNA control; ShRAPT, ShRAPTOR; 3-MA, 3-methyladenine; Wort, wortmannin; BafA1, bafilomycin A1; **, *p* < 0.01, ****, *p* < 0.0001

To evaluate if and how mTOR activity affects autophagy in TSC1 deficient cells in the presence of an mTOR inhibitor, TSC1 KO BMMϕ were treated with rapamycin (Rapa). Phosphorylation of S6 was inhibited upon rapamycin treatment in TSC1 KO BMMϕ and WT BMMϕ, which indicates that rapamycin suppresses constitutively active mTORC1 (Fig. 2a). However, TSC1 KO BMMϕ treated with various concentrations of rapamycin greatly reduced LC3B protein levels while the opposite was true for WT BMMϕ. Interestingly, p62 protein levels were decreased in both TSC1 WT and KO BMMϕ after 10 or 100 ng/ml rapamycin treatment (Fig. 2a). Similarly, two inhibitors of the Class III PI3K required for induction of autophagy, 3-methyladenine (3-MA) and wortmannin, greatly reduced LC3B protein levels in TSC1 KO BMMϕ while bafilomycin A1 treatment demonstrated the accumulation of LC3B-II, again confirming the presence of autophagic flux (Fig. 2b).

To directly examine the regulation of the autophagosome formation by mTORC1, we used a microscopy-based assay that quantified green fluorescent protein (GFP)-LC3 puncta formation in the absence and presence of rapamycin. LC3 was the first protein shown to specifically label autophagosomal membranes in mammalian cells, and subsequently EGFP-LC3 has become one of the most widely utilized reporters of autophagy [36]. Compared with the control, TSC1 KO BMMϕ showed higher numbers of puncta formation (Fig. 2c and d) without rapamycin treatment. However, TSC1 KO BMMϕ treated with rapamycin displayed a greatly reduced number of puncta while the same treatment increased the number of puncta in WT BMMϕ (Fig. 2c and d).

To further confirm this finding with genetic evidence, we generated a knockdown of RAPTOR, an essential component of mTORC1 complex [37], in TSC1 KO BMMϕ using shRNA. The knockdown of RAPTOR (TSC1 KO/RAPTOR KD) dramatically reduced LC3B protein and p-S6 levels in these cells (Fig. 2e). Taken together, the finding that the reduced activation of mTORC1 in TSC1 KO macrophages led to reduced autophagy rather than further increased autophagy, suggests that strong autophagy induction in the TSC1 KO macrophages may be used to degrade and recycle the cytoplasmic components and thereby to maintain the cellular homeostasis.

### Mycobacterial infection increased autophagy in TSC1 KO BMMϕ

**Fig. 3:**
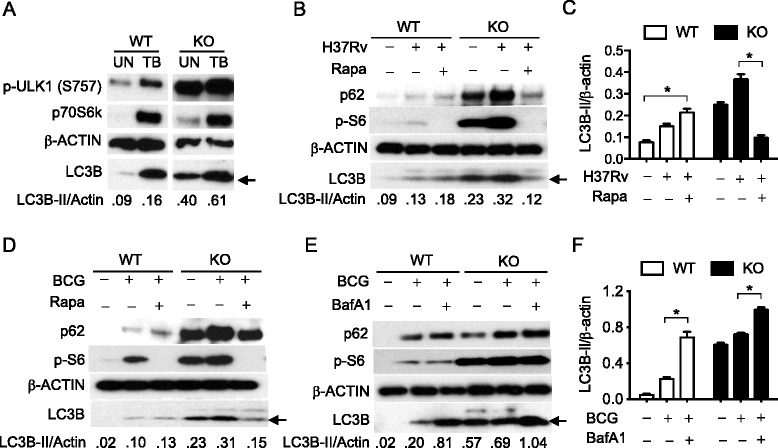
Mycobacterial infection further increased autophagy in TSC1 KO BMMϕ. **a** Tsc1 KO macrophages have mTORC1-dependent inhibition of ULK1 at S757. BMMϕ cells from either TSC1 WT or KO BMMϕ were left uninfected or infected with *M. tuberculosis* strain H37Rv (TB) (**a**, **b**, **c**) or BCG (**d**, **e**, **f**) at a MOI of 10 for 24 h and lysed for immunoblotting analysis with the indicated antibodies. TSC1 WT and KO BMMϕ were treated with 100 ng/ml rapamycin (**b**, **d**) or with 100 nM bafilomycin A1 (**e**, **f**) for 3 h and then lysed for Western blot analysis. **c**, **f** Densitometric quantification of LC3-II (*n* = 3) was normalized to total Actin in **c** and **e**. The arrow indicates LC3B-II. These experiments were repeated three times. BafA1, bafilomycin A1; BCG, *Bacillus Calmette*-*Guérin*; Rapa, rapamycin; TB, *M. tuberculosis* strain H37Rv, WT, Wild type; KO, knockout; UN, uninfected. *, *p* < 0.05; **, *p* <0.01

To examine how constitutively activated mTOR may affect autophagy during mycobacterial infection, we examined if activated mTORC1 phosphorylates its downstream substrates in infected TSC1 KO BMMϕ. Uninfected TSC1 KO macrophages showed an increase in phosphorylation of p70S6K and ULK1 at the mTORC1-dependent inactivating site S757 (Fig. 3a) compared with WT macrophages, as expected. Infection of the TSC1 KO macrophages with *M. tuberculosis* strain H37Rv further increased the phosphorylation of p70S6K and the accumulation of LC3B-II and p62 (Fig. 3a, b, and c). Similar results were observed during Bacillus Calmette–Guérin (BCG) infection (Fig. 3d, e, and f). Phosphorylation of S6, the target substrate of p70S6K, was also substantially increased with mycobacterial infections in both TSC1 KO and WT BMMϕ (Fig. 3).

To investigate whether mTOR inhibitor, rapamycin, could alter bacterial-induced autophagy, we treated both TSC1 WT and KO BMMϕ with rapamycin 1 h prior to bacterial infection with TB (Fig. 3b and c) and BCG (Fig. 3d). Surprisingly, rapamycin reduced LC3B and p62 proteins in TSC1 KO BMMϕ, but increased them in WT BMMϕ, when compared to their respective no-rapamycin controls. To examine infection inducible autophagic flux, BMMϕ from TSC1 WT and KO were infected with BCG with or without bafilomycin A1 (Fig. 3e and f). Bafilomycin A1 treatment significantly increased LC3B and p62 protein accumulation in the infected TSC1 KO BMMϕ. The findings of the further enhanced induced-autophagy and concomitant mTORC1 activation during mycobacterial infection as well as decreased LC3B-II levels upon rapamycin addition in the TSC1 KO macrophages suggest that an undiscovered mechanism might regulate autophagy when mTOR is constitutively activated.

### Increased p-AMPKα contributed to enhanced autophagy in TSC1 KO BMMϕ

**Fig. 4:**
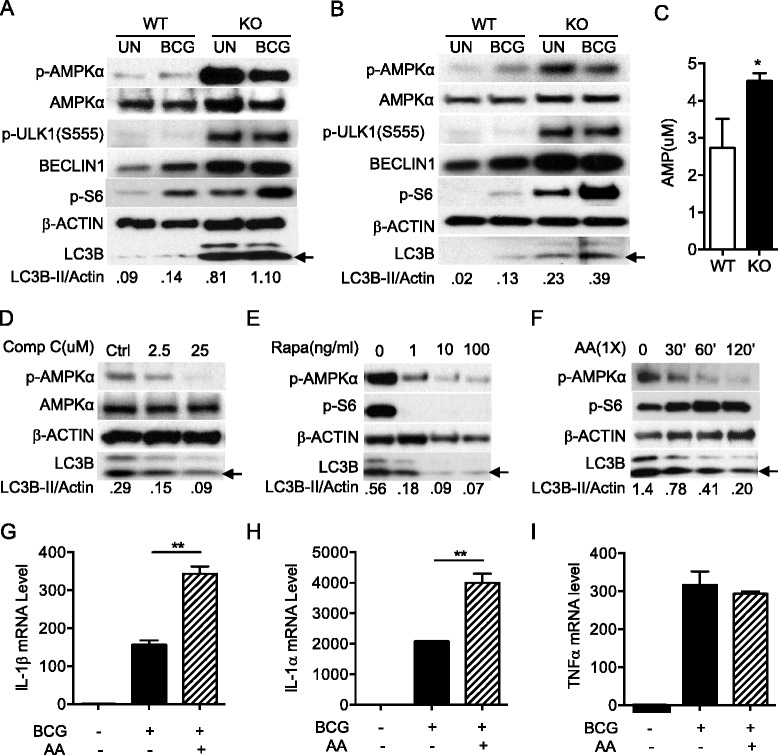
Increased p-AMPKα contributed to enhanced autophagy in TSC1 KO BMMϕ. TSC1 WT and KO peritoneal macrophages (**a**) or BMMϕ (**b**) were left uninfected or infected with BCG at a MOI of 10 for 24 h and Western blot analysis was performed against the indicated antibodies. **c** Quantification of AMP level was performed by LC-MC analysis in TSC1 WT and KO BMMϕ. **d** Compound C was added into TSC1 KO BMMϕ at the concentration of 0, 2.5, or 25 μM for 2 h. Cells were subjected to Western blot analysis. **e** TSC1 KO BMMϕ were treated with rapamycin at 0.1, 10, 100 ng/ml overnight. **f** Amino acids were added to TSC1 KO BMMϕ for 0, 30, 60 and 120 min. **g**, **h**, and **i** TSC1 KO BMMϕ were left uninfected or infected with BCG or BCG plus amino acids for 4 h and then lysed with Trizol reagent for qRT-PCR. IL-1β (**g**), IL-1α (**h**), and TNFα (**i**) mRNA levels in TSC1 KO BMMϕ are shown. Each experiment was repeated at least twice. Densitometric quantification of LC3-II (*n* = 3) was normalized to total Actin. The arrow indicates LC3B-II. AA, amino acids; BCG, *Bacillus Calmette*-*Guérin*; Comp C, compound C; KO, knockout; Rapa, rapamycin; WT, Wild type. *, *p* < 0.05; **, *p* < 0.01

Based on the *ex vivo* studies, we hypothesized that sustainably active mTOR may deplete energy or amino acids in TSC1 KO BMMϕ, which in turn would induce autophagy by other autophagy initiation pathways to overcome starvation. AMPK, activated during low nutrient conditions, directly phosphorylates ULK1 at multiple sites including Ser317, Ser555, and Ser777 (17,18). Conversely, mTOR, which is a regulator of cell growth and is an inhibitor of autophagy, phosphorylates ULK1 at Ser757 and disrupts the interaction between ULK1 and AMPK (17). It is known that autophagy induced by energetic stress requires AMPK, which directly activates ULK1 through phosphorylation at Ser555 [2, 33, 38]. To explore the underlying mechanisms by which constitutively active mTOR induces autophagy, cell lysates from TSC1 WT and TSC1 KO BMMϕ (Fig. 4a) and from TSC1 KO peritoneal macrophages (Fig. 4b) were examined for p-AMPKα, p-ULK1 (Ser555), Beclin1, and LC3B protein levels. Activated ULK1 (Ser555) induces autophagy by phosphorylating Beclin-1 following amino acid starvation [20, 39]. Indeed, phosphorylated AMPKα, ULK1 (Ser555), and Beclin1 protein levels were higher in TSC1 KO BMMϕ than WT BMMϕ before or after BCG infection (Fig. 4a, b). TSC1 KO macrophages showed an increase in ULK1 phosphorylation at the mTORC1-dependent inactivating site S757 (Fig. 3a) as expected. AMP concentration determined by LC-MS analysis was significantly higher in TSC1 KO BMMϕ than WT BMMϕ (Fig. 4c). Together, these data support that sustainably active mTOR causes energy depletion in TSC1 KO BMMϕ, which in turn increases p-AMPKα and subsequently activates ULK1 (Ser555) to initiate autophagy.

To confirm that LC3B up-regulation in TSC1 KO BMMϕ was AMPK dependent, compound C (a pharmacological AMPK inhibitor) [40], was used to treat TSC1 KO BMMϕ for 1.5 h. As showed in Fig. 4d, compound C reduced both p-AMPKα level and LC3B protein, which indicates that AMPK activity regulates autophagy induction in TSC1 KO BMMϕ cells. Furthermore, rapamycin inhibited the elevated p-AMPKα and reduced LC3B protein level in KO BMMϕ (Fig. 4e). We tested if the TSC1 defect associated phenotypes can be reversed by addition of amino acids or glucose. The addition of amino acids (Fig. 4f) into TSC1 KO BMMϕ medium reduced both LC3B and p-AMPKα expression levels and activated mTOR, indicating that sustainably activated mTOR leads to the depletion of amino acids in TSC1 KO BMMϕ. Glucose supplementation did not change the LC3B level (data not shown).

Dual roles of autophagy in controlling bacterial burden and suppressing inflammation with features of a Th17 response including neutrophilic infiltration, tissue necrosis, and organ damage, have been previously reported [41]. As such, we were interested in evaluating the inflammatory cytokine levels in KO BMMϕ. Total RNA was isolated from TSC1 KO BMMϕ left uninfected or infected with BCG with or without amino acids. BCG infected TSC1 KO BMMϕ supplemented with amino acids displayed increased IL-1α and β mRNA transcripts compared to the cells infected with BCG without amino acid supplementation (Fig. 4g and h). TNFα mRNA levels were not affected in the same infected cells (Fig. 4i). These results suggested that amino acid depletion caused by constitutive activation of mTORC1 activates p-AMPKα and p-ULK1 (Ser555) to promote autophagy in order to keep homeostasis in TSC1 KO BMMϕ.

### TSC1 KO mice infected with *M. tuberculosis* H37Rv showed reduced bacterial burden

**Fig. 5:**
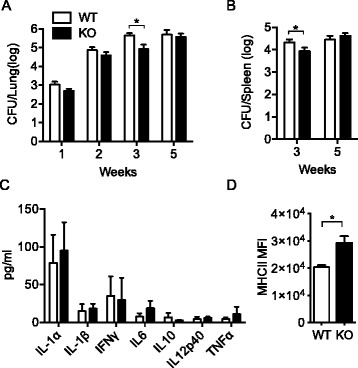
Infection of TSC1^f/f^ LysM-Cre^+^ mice with *M. tuberculosis* resulted in lower mycobacterial counts at the time of the arrival of adaptive immune cells. Both TSC1^f/f^ LysM-Cre^+^ and LysM-Cre^-^ mice were aerogenously infected with *M. tuberculosis* H37Rv with 200-300 bacilli per lung. Bacterial burdens were measured by CFUs in the lungs (**a**) at 1, 2, 3, and 5 weeks post-aerosol infection and in the spleens (**b**) at 3 and 5 weeks post-aerosol infection (*n* = 4 per time point). **c** Cytokine secretion from lung homogenates was determined by Luminex at 3 weeks post-aerosol infection. The data were shown as mean ± SD is shown (*n* = 4). **d** Flow cytometry analysis of MHCII expression in TSC1 WT and KO macrophages at 3 weeks after *M. tuberculosis* infection is shown. MFI ± SD is shown (*n* = 4). MFI = mean fluorescence intensity. *, *p* < 0.05

Host cells utilize autophagy pathways in their defense against pathogens. Autophagy limits mycobacterial replication by targeting phagosomes containing bacteria for fusion with lysosomes [41, 42]. We further investigated how mice with basally enhanced autophagy due to TSC1 deficiency in macrophages survive *M. tuberculosis* H37Rv infection using TSC1^f/f^ LysM-Cre^+^ or littermate control mice. Statistically significant reduction of bacterial burden was observed in both lungs (Fig. 5a) and spleens (Fig. 5b) from TSC1^f/f^ LysM-Cre^+^ mice 3 weeks post-infection compared to WT mice, suggesting that greater autophagy induction in TSC1 deficient macrophages may cause killing of infecting bacilli. However, there was no statistical difference in lung and spleen CFUs between TSC1^f/f^ LysM-Cre^+^ and TSC1^f/f^ LysM-Cre^-^ mice at 5 weeks post-infection.

Luminex data showed that there was no significant difference in IL-1α and β secretions in lung homogenates between TSC1 WT and KO mice; there was also a comparable level of proinflammatory cytokines such as IFNγ, IL-6, IL10, IL12p40, and TNFα in lung homogenates (Fig. 5c). Major histocompatibility complex II (MHCII) expression in spleen macrophages was higher in TSC1^f/f^ LysM-Cre^+^ than TSC1^f/f^ LysM-Cre^-^ mice at 3 weeks after *M. tuberculosis* H37Rv infection (Fig. 5d), which is consistent with other reports [43–47] and further supports our finding that there is ongoing autophagy in TSC1 KO macrophages. Together, TSC1^f/f^ LysM-Cre^+^ mice were able to temporarily contain *M. tuberculosis* H37Rv infection at the time of recruitment of *M. tuberculosis* specific CD4^+^ T cells to the site of infection, possibly due to enhanced autophagy.

## Discussion

The inhibitory function of mTORC1 in autophagy is well established [31, 48] as mTORC1 activity reflects cellular nutritional status [49]. Our initial hypothesis was that constitutive mTORC1 activation in macrophages could suppress autophagy, as shown in other cells. However, our in vitro and in vivo experiments generated the opposite results and TSC1 deficient macrophages had higher basal levels of LC3B expression compared to wild type controls. Our data are similar those derived from experiments using *Tsc1*/*Tsc2*-deficient neurons which were shown to have increased autolysosome accumulation and autophagic flux despite mTORC1-dependent regulation of ULK1 [33]. Both in the *Tsc2*-knockdown neurons and in the brains of Tsc1 conditional mouse models, AMPK activation is the dominant regulator of autophagy. However, unlike in the TSC1 KO macrophages, the baseline number of autophagosomes in TSC1/2 deficient neurons was unchanged despite ULK1 (S757) inhibition, and the accumulation of autolysomes was rapamycin-insensitve. In contrast to TSC1/2 KO macrophages and neurons, it was previously reported that Tsc1/2-deficient mouse embryonic fibroblasts (MEFs) have reduced autophagy via mTORC1-dependent phosporylation of ULK1 at S757 [20]. Similarly, sustained activation of mTORC1 in skeletal muscle inhibits constitutive and starvation-induced autophagy and causes a severe, late-onset myopathy [26]. Furthermore, due to constitutive mTOR activation, the resulting impairment of autophagy sensitizes TSC2-null cells to cell death under stress [50]. When starved, TSC1-deficient T cells show a comparable level of autophagy compared to wild type T cells, indicating that the increased mTORC1 activity does not inhibit autophagy in T cells [51]. Such variations in mTORC1 dependent autophagy regulation could be attributed to the different cells examined.

In order to demonstrate the active autophagic flux in TSC1 KO macrophages, we also measured p62 levels as an important indicator of autophagy-mediated degradation of cellular contents. p62 is rapidly degraded during autophagy and analysis of its intracellular level by Western blotting is used routinely to measure the autophagic flux in response to pro-autophagic stimuli [8]. However, in our study, p62 protein levels were higher in TSC1 KO BMMϕ than TSC1 WT BMMϕ and p62 accumulation was sensitive to rapamycin (Figs. 2a and 3b). We believe that that the concomitant elevation of p62 and LC3B-II in TSC1-KO macrophages results from the inability of turnover to keep pace with increased autophagosome formation, rather than reduction in autophagosome turnover. When TSC1/TSC2 are disrupted, mTORC1 is consistently activated to phosphorylate several downstream regulators for protein synthesis, lipid synthesis, or glycolytic metabolism, leading to energetic stress. These energetic stresses then activate AMPK, which is a key energy sensor and regulates cellular metabolism to maintain energy homeostasis [52]. Activated AMPK inhibits mTORC1 through phosphorylation of TSC2 and Raptor and at the same time phosphorylates Ulk1 on Ser555 and then initiates autophagy [20, 53]. Thus, in TSC1-KO macrophages, autophagic machinery is overwhelmed with many autophagosomes accumulated by AMPK-dependent autophagy activation due to energetic stress signal. Upon treatment of compound C (Fig. 4d), rapamycin (Fig. 4e), or amino acid (Fig. 4f), the mTORC1 will be inhibited and cellular stress will be reduced, which results in lower level of autophagy activation. Similar results were also observed in TSC1/2 KO MEFs and neurons [33], which have p62 accumulation that was mTORC1 dependent.

Our data also showed that TSC1^f/f^ LysM-Cre^+^ mice have less or similar bacterial burdens in lungs and spleens of mice aerosol infected with *M. tuberculosis* H37Rv compared to wild type mice (Fig. 5). In contrast, mice with autophagy defects (ATG5 KO) showed increased bacterial burden and inflammation in lungs [54]. Thus, consistent with the enhanced autophagy phenotype shown in TSC1 deficient BMMϕ, our in vivo studies indicated the increased autophagy induction, not inhibition, in TSC1 KO mice, which was also supported by a similar level of inflammatory cytokines and enhanced MHCII level in TSC1 KO mice. These data implicate the use of novel autophagy inducers as a therapeutic strategy for TB, especially for drug resistant TB. However, direct in vivo studies with autophagic protein (*atg*) deficiency in TSC KO macrophages will confirm the result that *M. tuberculosis* containment and increased MHCII levels in TSC1 KO mice are the consequence of increased autophagy.

In summary, our data indicate that constitutively activated mTORC1 in TSC1 deficient macrophages increases autophagy through AMPK-dependent regulation of ULK1, likely to balance energy homeostasis. These findings highlight the complex regulatory network that modulates energy metabolism in cells. The fact that mTORC1 deregulation is linked to several human diseases, such as type 2 diabetes, cancer, obesity and neurodegeneration, highlights the importance of this signaling pathway in the maintenance of cellular homeostasis [55]. Our findings are thus relevant to the study of these diseases and may provide novel insight for the treatment of many human diseases.

## Conclusions

Metabolic homeostasis is orchestrated by mTORC1 through the promotion of biosynthetic pathways and the repression of catabolic autophagy in response to energy and amino acid sufficiency. Collectively, the regulation of these processes is strictly determined by energy and amino acid sensing pathways within the cell [31, 49]. However, how constitutive mTORC1 activation affects autophagy is still controversial. In contrast to the previous studies that the TSC1-TSC2 complex is a critical negative regulator of mTORC1 and that TSC1/2 deficient cells have reduced autophagy via mTORC1-dependent inhibition and phosphorylation of ULK1 at S757, we observed that TSC1 deficient macrophages had higher basal and infection induced autophagy compared to wild type controls. This autophagy dysfunction was mediated through AMPK-dependent phophorylation of ULK1 at Ser555 concomitant with mTORC1 inhibition of ULK1 at Ser757. In fact, our results demonstrate that TSC1 KO macrophages have increased AMP levels suggesting energetic stress. These results collectively suggest that AMPK activation in TSC1 KO macrophages is a positive feedback mechanism on autophagy to prevent long-term cellular stress and maintain cellular homeostasis.

## Methods

### Mice and reagents

TSC1^f/f^-ERCre^+^ mice were previously reported [27, 56]. LysM-Cre^+^ mice were purchased from Jackson Laboratory. *M. bovis* (Bacillus Calmette-Guérin or BCG) was obtained from the laboratory of Dr. William Jacobs (Albert Einstein College of Medicine). Virulent *M. tuberculosis* H37Rv (ATCC, 25618D-2) was purchased from ATCC. Rapamycin was obtained from Enzo Life Science (Enzo Life Science, A275). Bafilomycin A1 (B1793), 3-methyladenine (3-MA, M9281), and wortmannin (W1628) were purchased from Sigma-Aldrich. Compound C (Millipore, 171,260) was obtained from Millipore (Billerica, MA).

### *M. tuberculosis* H37Rv in vivo infection and CFU determination

TSC1^fl/fl^ LysM-Cre^+^ and TSC1^fl/fl^ LysM-Cre^-^ mice were infected with H37Rv via aerosol infection (200-300 CFUs) as previously described [57]. The retained dose was determined by lung necropsy in four mice sacrificed 24 h after exposure. At specified time points, lungs and spleens removed from the mice were placed in a WhirlPak bag containing 4.5 mL of PBS and homogenized by manually rolling a pipette on the bag. Defined volumes of the neat homogenate and serial dilutions were plated on 7H10 agar plates. The plates were incubated for 4 weeks at 37 °C. The upper limit and lower limit for colony counting was 300 and 30 colonies per plate, respectively. The total CFU for each organ was calculated by multiplying CFU counts by their respective dilution factors.

### Bone marrow derived macrophage (BMMϕ) development and mycobacterial infection

Bone marrow cells from femurs and tibias were flushed and plated into petri dishes containing RPMI 10 (RPMI 1640 medium supplemented with 10 % FBS, 100 U/ml penicillin, 1000 U/ml streptomycin, and 20 mM L-glutamine) containing 15 % L929 cell conditioned medium. After 2–3 days of culture at 37 °C in a CO_2_ incubator, non-adherent cells were transferred to new plates with fresh medium for an additional 3–5 days before they were used for experiments. To remove TSC1 protein in BMMϕ in vitro, 4-Hydroxytamoxifen (Sigma-Aldrich, H7904) at 2.5 mM was added into L929 conditioned culture medium for 2–3 days before use. For the resident peritoneal macrophage preparation, macrophages were washed from the peritoneal cavity with 5 ml of 1x PBS. After centrifugation at 1500 rpm for 5 min, the cell pellets were resuspended in complete medium (RPMI1640 containing 10 % FBS and antibiotics) and plated in a 12-well plate. After 2–3 h, non-adherent cells were removed and freshly prepared complete medium was added with the indicated stimuli. For mycobacterial infection within 3 h, cells were washed twice with 1x PBS after infection and then harvested for indicated analysis. For mycobacterial infection beyond 3 h, cells were washed twice with PBS 3 h after infection, and then grown in fresh complete medium supplemented with 50 μg/ml of gentamycin for indicated periods.

### Immunofluorescence microscopy

TSC1 WT and KO BMMϕ were grown on chamber slides (BD Discovery Labware, 154534) in 10 % FBS RPMI 1640 for 24 h at 37 °C. The cells were treated with or without rapamycin at 20 ng/ml overnight. The cells were washed with PBS twice, and then were fixed in 4 % paraformaldehyde/PBS for 10 min, permeabilized with 0.2 % Triton X-100/PBS for 10 min, and pre-blocked in 5 % BSA/PBS overnight. The slides were then incubated with anti-rabbit LC3B antibody (Cell Signaling Technology, 2775) that was diluted at 1/100 in blocking solution for 2 h, washed three times with PBS, and incubated with Alexa fluor 488-conjugated anti-rabbit IgG Ab (Invitrogen, A11034). One hour after additional washes, the slides were mounted with DAPI containing anti-fade solution (Vector Laboratories, INC., H-1200). Images were taken on a Nikon TE 2000 immunofluorescent microscope and analyzed with NIS Elements software (1,000x magnification). Autophagic puncta were randomly counted for 20 cells in each group and averaged.

### ELISA/Luminex

BMMϕ (3 × 10^5^) cells were plated into each well of 24 well-plates and allowed to adhere overnight (Becton Dickinson Labware, 3574). Both TSC1 WT and KO BMMϕ were infected with BCG or *M. tuberculosis* H37Rv at an MOI of 10 for indicated periods. The culture supernatants were harvested to detect IL-1β cytokine levels and were determined by using a commercial ELISA kit (Biolegend, 432606) according to the manufacturer’s instructions. These cells were then harvested for total RNA isolation. For Luminex assay, the Mouse Cytokine/Chemokine Magnetic Bead Panel kit was purchased from EMD Millipore Corporation (Billerica, MA). The procedure followed the manufacturer’s instructions using the Bio-Plex 200 System (Bio-rad).

### qRT-PCR

Total RNA was prepared by using TRIzol® reagent (Invitrogen, 15596-018). Reverse transcription was performed using iScript Reverse Transcriptase (Bio-Rad, 170-8840). SYBR green real-time PCR was conducted using iQ™ Supermix (Bio-Rad, 170-8880). All reagents were used according to the manufacturer protocol. mRNA expression level is defined as fold change over control (arbitrarily as 1). The primer pairs were used as follows: β-Actin: forward 5’ TGTCCACCTTCCAGCAGATGT 3’ and reverse 5’ AGCTCAGTAACAGTCCGCCTAGA 3’; TNFα: Forward 5’CCCCAAAGGGATGAGAAGTT and Reverse 5’ CACTTGGTGGTTTGCTACGA; IL-1α: Forward 5’CGTCAGGCAGAAGTTTGTCA and Reverse 5’ TTAGAGTCGTCTCCTCCCGA; IL-1β: Forward 5’CTCATCTGGGATCCTCTCCA and reverse 5’ TGTCAAAAGGTGGCATTTCA.

### Western blot

TSC1 WT and KO BMMϕ (3 × 10^5^ cells/well) were seeded into 12-well plates in RPMI complete medium without L929 conditional medium overnight. Cells were infected with the indicated bacteria at an MOI of 10. After 3 h post infection, cells were washed once in PBS followed by addition of complete medium supplemented with 50 ug/ml gentamycin (Invitrogen, 15750-060). Cells were cultured until the indicated time points. Cells were washed once in PBS and lysed in 1 % Triton-100 lysis buffer (1 % Triton-100, 150 mM NaCl, 50 mM Tris, pH 7.4, 1 mM EDTA) with protease inhibitor tablet and phosphatase inhibitor cocktails (Sigma, S8820). Protein concentrations were determined using a Bio-Rad Protein Assay at OD600 nm (Bio-Rad, 500-0006). Equivalent amounts of protein for each sample were subjected to SDS-polyacrylamide gel electrophoresis and transferred onto methanol equilibrated PVDF membrane (BIO-RAD Laboratories, 162-0177). After blocking with 5 % nonfat dry milk/PBS, the membranes were incubated with a primary antibody overnight at 4 °C, and washed with PBST (100 mM Tris, pH 7.5, NaCl 0.9 %, Tween 0.1 %). Secondary antibody was added for 45 min at room temperature, and the membrane was washed with PBST three times. Protein bands were visualized by ECL (Perkin Elmer Life Sciences, NE104001EA). The following antibodies were used for immunoblotting: Rabbit anti-LC3B (2775), p-S6 (Ser240/244) (4857), p-ULK1 (Ser757) (6888) or (Ser555) (5869), p-AMPKα (T172) (2535), Beclin1 (3738), ATG5 (2630), p62 (5114) from Cell Signaling Technology, and mouse anti-β-actin (Sigma-Aldrich, A5441). Films were scanned and ImageJ software was used to quantify band intensities. As described in Individual figures, band intensity of LC3B-II was related to the level of β-actin.

### Flow cytometry

Cells were stained with the indicated antibodies in FACS buffer (PBS containing 2 % FBS) at 4 °C for 30 min. Cells were pelleted and washed once with FACS buffer and resuspended in 4 % paraformaldehyde at least 2 h before analysis. Fluorescently conjugated antibodies used were APC-anti-F4/80 (123116), PE/Cy5-anti-Gr1 (108410), PE/Cy7-anti-CD11b (101216), and PE-anti-CD45 (103106) from Biolegend. Data were collected using a BD FACS canto or LSRII and analyzed with FlowJo (Tree Star).

### AMP quantitation

Approximately 30 million BMMϕ cells from TSC1^f/f^-ERCre^+^ or TSC1^f/f^-ERCre^−^ mouse were plated into one 10 cm petri dish in 15 % L929 conditional medium in triplicate. After 24 h, cells were washed twice with the warmed 1x PBS, scraped in the same PBS, and spun at 2000 rpm for 5 min at 4 °C. The cell pellets were stored at -80 °C for future use. The LC-MS/MS method was described previously [58]. The sample processing was performed in the DMPI Metabolomics Laboratory, Duke University.

### ShRNA transduction

ShRaptor and ShLuc control were purchased from Addgene (21339). 293FT cells were transfected using the Fugene 6 Transfection Reagent (Promega Corporation, E269A). The viral supernatants were collected 48 h after transfection and frozen at -80 °C for future transduction. For infection, 2 × 10^5^ cells in 1 mL of RPMI 10 were mixed with 0.5 mL of viral supernatant in a 6-well plate, along with 5ug/mL of polybrene (EMD Millipore, TR-1003-G). Cells were centrifuged at 2500 g for 1.5 h at room temperature. Fresh complete medium was added to the cells 7 h after infection. After 24 h of infection, cells were treated with puromycin (Sigma, P9620) at 2 μg/ml for 3-4 days.

### Statistical analysis

*P* values were calculated using the student’s *t*-test or ANOVA analysis. *P* values of less than 0.05 or 0.01 were considered significant or very significant, respectively.

## Abbreviations

3-MA, 3-methyladenine; 4EBP1, eukaryotic translation initiation factor 4E-binding protein 1; AA, amino acid; AMP, adenosine monophosphate; AMPK, AMP-activated protein kinase; ATG, autophagy-related gene; BafA1, BCG, Bacillus Calmette-Guérin; KO, knock out; CFU, colony forming unit; LC3, microtubule-associated protein 1A/1B-light chain 3; MEF, mouse embryonic fibroblasts; MFI, mean fluorescence intensity; mTOR, mechanistic target of rapamycin; mTORC1, mechanistic target of rapamycin complex 1; p70S6K, 70 kDa ribosomal protein S6 kinase 1; Raptor, regulatory-associated protein of mTOR; S6, ribosomal protein S6; TSC, tuberous sclerosis complex; ULK1/2, UNC-51 like autophagy activating kinase 1/2; Wort, wortmannin; WT, wild type
